# Some Remarks on the Treatment of Dysenteric Diarrhea

**Published:** 1901-07

**Authors:** George Torian

**Affiliations:** Cody, Va.


### SOME REMARKS ON THE TREATMENT OF DYSENTERIC DIARRHEA.

BY GEORGE TORIAN, M.D.,

CODY, VA.

Frequently in the treatment of diarrheal affections I have met with cases in which the ordinary remedies proved of no avail, and then, after searching the text-books and journals, I was so fortunate as to find some happy combination which fulfilled the special indications in that particular instance. Hence, I have been led to believe that there is more real practical help from an interchange of views, and from reported clinical observations on therapeutic subjects in the medical journals, than from any other source of information. It was in this way that my attention was drawn to tannopine, and I have always considered this a fortuitous circumstance, for this drug has given me more perfect results in the cases in which it is indicated than any other which I have yet tried.

Instead of discussing the special indications of tannopine, I have thought it preferable to cite the histories of a number of cases which will best serve to illustrate the conditions in which it is particularly applicable. As will be noted, these cases comprise various types of diarrhea and dysentery, such as are so frequently observed during the warm season of the year.

CASE I.—Mrs. H. suffered from an attack of cholera morbus in July, 1900, due to the heat, the fatigue incident to a church fair, and to errors in diet customary on such occasions. Being hastily summoned, I reached her residence after sunrise, and found her collapsed from profuse vomiting and discharges from the bowels, as well as suffering from excruciating pain and tenesmus before every evacuation. Her husband stated that she had had a movement every thirty minutes since the middle of the night, accompanied with a distressing retching and nausea. I ordered heat to be immediately applied to the abdomen, and also frequent small doses of ipecac with an anodyne. Several hours later, when nausea had subsided, I administered powders containing tannopine, 3 grains; bismuth subnit., 3 grains; mercury with chalk, 1 grain, and Dover's powders, 2 grains; one of these was administered every fifteen minutes until four had been taken, then every hour until the discharge was checked. This was followed the next morning by a full dose of castor oil. On calling the next day I found the patient up and dressed. She stated that the diarrhea ceased after the third dose, and that except for some soreness, she felt perfectly comfortable. A strict diet was enjoined for the first few days, and no further trouble experienced.

CASES II., III. and IV. were similar, the patients being children, varying in age from six to twelve months, who for several days had had bloody, greenish mucoid discharges, and suffered considerable pain before each movement; the temperature was elevated in each instance. I prescribed tannopine, 1 grain; pulv. ipecac, $\frac{1}{8}$ grain; mercury with chalk, $\frac{1}{2}$ grain; bismuth subnit., 2 grains; one powder being given every half-hour until four had been taken, and then hourly. A dose of castor oil was administered on the following morning, withholding all food until the oil had acted. Under this treatment the patients recovered promptly, only two visits being necessary.

CASE V.—B. C., aged twenty-two, farmer; was attacked on the second Sunday of August with violent headache, general aching in the limbs, loss of appetite, and a feeling of malaise, but did not seek medical advice until the following Wednesday. I saw him at 9 o'clock in the morning, and found the following condition: The tongue was heavily furred with a brown coating; the abdomen distended and tympanitic, and very sensitive to the touch; the urine scant and high colored, producing burning when voided; the limbs and lower part of the back were painful and sore. There was headache and incessant thirst; rose-colored spots were present on the face and body; the bowels moved about every hour; the evacuations being of the character of pea soup, and having a musty odor; temperature 102.2°. I gave calomel, 6 grains; podophyllin, ¼ grain, and acetanilid, 5 grains. At my next visit at four P.M. I found his condition unchanged, except that the temperature had risen to 104°. The morning prescription was renewed, omitting the calomel, which was given separately with soda in one half-grain pellet hourly. On Thursday and Friday his condition remained unchanged, except that the head trouble was much worse, his father stating that he had to hold him in bed for two nights previous for a number of hours, as the patient was perfectly wild and delirious. Owing to this change for the worse, I decided to adopt a different course of treatment. Up to now I had been careful to keep my opinion to myself, but on Friday I was asked to diagnose the case so emphatically that I was compelled to state that if it was not genuine typhoid fever, I could not say exactly what it was. They then told me that a neighbor of theirs had declared it a very malignant case of typhoid, and had expressed the opinion that the patient would certainly die. In order to exert a direct effect upon the bowel complications, I prescribed the following: Tannopine, 10 grains; bismuth subnit., 30 grains; Dover's powder, 15 grains; mercury with chalk, 10 grains. Divide into six powders, one to be given at first every hour, for three hours, and then every two hours. I also gave Fowler's solution, 5 drops, three times daily, and the following: spirits of turpentine, 3 drops; spirits of nitrous ether, 15 drops, every four hours; and tincture of veratrum viride, 6 drops, every four to six hours, until the body surface became moist. A cloth saturated with oil of turpentine was ordered to be kept on the abdomen continuously. On Saturday morning I found the patient sitting up on a pallet placed near the door, and upon inquiry he stated that he felt tolerably well. Considering his previous condition I thought he must be delirious, but upon using the thermometer found his temperature normal. The tongue had changed greatly; the abdomen was no longer tympanitic, and only slightly sore; the kidneys were acting normally; the headache had disappeared, and the bowels had moved only twice since he took the fourth dose of the powders, the last movement being that morning, and quite normal. The patient had rested well the night before, and was really hungry. His recovery was uneventful and complete in about two weeks. While I do not claim that this was absolutely a case of typhoid fever, since this would be to invite criticism, I would say that it manifested every symptom belonging to this malady. It is also my belief, that tannopine contributed to the rapid recovery by reason of its special effect upon the intestinal condition.

CASE VI.—This patient had been sick with undoubted typhoid fever for three weeks, the discharges being of a dysenteric character. For this reason I put him on the same treatment as employed in the preceding case, with the result that in forty-eight hours the bowel symptoms were completely relieved, and the temperature normal. The patient made a complete though slow recovery, owing to the marked emaciation present.

CASE VII.—M. R., aged fifteen months; was attacked with thrush, which persisted for three weeks, the infection extending along the entire alimentary tract, and involving even the anus. She had a griping movement with tenesmus every half to one and one half hours, and when this was checked she was seized with vomiting and high fever. My treatment consisted of tannopine, 20 grains; pulv. ipecac, 3 grains; bismuth subnit., 20 grains; mix and divide into eighteen powders. Sig.: One every two hours.

This was followed by turpentine, 2 drops; with a purgative dose of castor oil as soon as the bowels had become easier and the stomach would tolerate it. In two days the dysenteric symptoms subsided, and I then ordered turpentine, two drops, to be given in a teaspoonful of castor oil, thrice daily, before meal-time, and an emulsion of cod-liver oil with wine of pepsin being given immediately after meals. Tepid baths were also recommended. This treatment was followed by rapid improvement and recovery was complete in about eight weeks.

CASES VIII. and IX. comprised patients suffering with dysentery, with bloody and mucous stools and severe tenesmus. In the one, a child two years old, who had been having sixteen to eighteen movements in twenty-four hours, was rapidly relieved by powders of tannopine, 1 grain; mercury with chalk, 1 grain; given every two hours, and followed by a dose of oil the next morning. The other case was that of a feeble old woman, who derived equally good results from the same treatment. The bowels which had been acting fifteen times in twenty-four hours, moved only twice a day after five days' treatment, and in other respects she was practically well.

